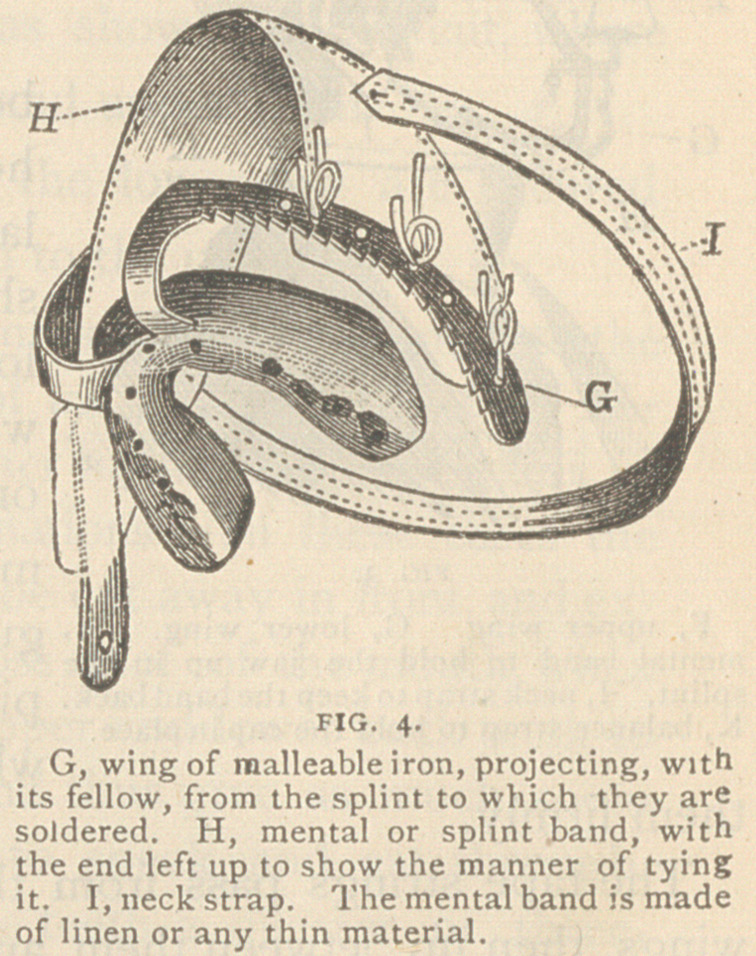# Treatment of Fracture of the Jaw, with Critical Remarks

**Published:** 1880-12

**Authors:** Thomas Brian Gunning

**Affiliations:** New York


ARTICLE III.

### TREATMENT OF FRACTURE OF THE JAW, WITH CRITICAL REMARKS.

BY THOMAS BRIAN GUNNING, D. D. S., NEW YORK.

Without full assurance of the efficiency of these appliances, prognosis in fractures of the maxillæ must continue uncertain, and the most experienced surgeon might find his patient had passed into the care of another whose use of a splint resulted in a successful cure. Or worse still, being misled in respect to the splints, keep his unfortunate patient suffering under the old treatment for months, and then perhaps deformed for life.

M. Malgaigne says:

* "Our prognosis should have reference to several points, viz: the favorable or unfavorable termination of the case; its simple or complicated course; the influence of each complication; the duration of the treatment; and lastly, the result as to the functions of the limb." Then after two pages of important suggestions, he says:

† "On the whole, then, to form a judicious prognosis, the surgeon should take into account the age of the patient, the sex, the state of strength or debility, of health or sickness, the circumstances of the fracture, as regards its seat, its nature, its recent or ancient date, its complications; and lastly, the plan of treatment already pursued, as well as that proposed for the future."

Sir James Paget says: ‡ "Doubtless the conditions necessary to the normal nutrition of parts are very many; but the chief of them are these four:

1. A right state and composition of the blood or other nutritive material.
2. A regular and not far distant supply of such blood.
3. (At least in most cases) a certain influence of the nervous system.
4. A natural state of the part to be maintained."

Sir James Paget in referring to the deprivation of nerve force, says: § "A man with nearly complete paraplegia, and distorted feet, the

*Op. Cit., p. 140. † Op. Cit., p. 142.

‡Lectures on Surgical Pathology, p. 11.

§Op. Cit., p. 34.

consequence of injuries of the spine, in whom some tendons were subcutaneously divided and appeared to be healing; but a bandage being applied rather tightly, sloughing ensued at the instep, on which the chief pressure fell, and extended widely and deeply to the ankle joints."

Again, in asking through what class of nerves is the nutritive process influenced? He says:

*" Indirectly, it is certain that the motor or centrifugal nerves may influence it, for when these are paralyzed the muscles they supply will be inactive, and atrophy will ensue, first in these muscles, then in the bones, (if a limb be the seat of the paralysis,) for the bones in their nutrition observe the example of their muscles; and finally, the want of energy in the circulation, which is in some measure dependent on muscular action, will bring about the atrophy of the other tissues of the part."

Further, in his approximate estimate of the time taken by the several parts of the reparative process in fractures of adult human bones, he says:

†To the second or third day after the injury, inflammation in and about the parts; thence to the eighth or tenth, seeming inaction, with subsidence of inflammation; thence to about the twentieth, production of the reparative material, and its gradual development to its fibrous or cartilaginous condition; thenceforward its gradual ossification, a part of the process which is, however, most variable in both its time of commencement and its rate of progress, and which is, probably, rarely completed before the ninth or tenth week; although the limb may have long previously recovered its fitness for support or other use. From this time the rate of change is so uncertain, that it is impossible to assign the average time within which the perfection of the repair is, if ever, accomplished."

The Hon. William H. Seward, Secretary of State, met the injury on April 4th, 1865, through jumping from his barouche while the horses were running away. The heel of his boot caught and threw him over so that his right arm struck the ground and his face was bruised. He was insensible from concussion for a short time, and on examination, his right arm was said to be broken. The lower jaw was also found to be fractured on the right side, as the edges hurt his

*Ibid, p. 35. †Op. Cit., p. 193.

tongue. The attempts to hold the fragments of the jaw in place, failed entirely; and on the tenth day I was called to the case.

The patient, 65 years old, had been without teeth in the upper jaw for several years and the alveolar process, which is only an appendage of the teeth, had with much of the gum been absorbed, so that the roof of the mouth was flat and very far from the lower front teeth. Yet attempts had been made repeatedly to hold the fragments of the jaw together by drawing the teeth up to the roof of the mouth by means of bandages around the chin, face and head; on the principle practiced by Hippocrates and Soranus of making the upper teeth a splint for the broken lower jaw, except that this patient had no upper teeth for the lower ones to rest against, consequently, the fragments of the bone and the adjoining soft parts were so distorted as to cause great pain. In the language of the patient: "Coals of fire could not have hurt me more, and when I could bear it no longer I would tear the bandages off."

Yet the patient had an upper set of artificial teeth, which could have been placed in the mouth so that the lower teeth would have had a resting place when the bandage was around the jaw and head. This would have held the parts less displaced than without the plate; and if the four front teeth had been removed from the plate, the space left between it and the edges of the lower teeth would have sufficed to allow food, etc., to enter the mouth. Had any difficulty prevented the surgeons from doing this, Dr. H. N. Wadsworth,* dentist of Washington, was but a few minutes distant, and as he was the first to use gutta-percha on fractures of the jaws; having applied it on the teeth in 1847, and outside the mouth around the lower jaw in 1850, he could have arranged matters temporarily, at least, so that the patient would have suffered much less, the parts have been less injured, and the patient's life less jeopardized.

Ligatures had also been used on the teeth to hold the fragments together, but I saw but one, this was when I examined the jaw on April 16th, it was of wire which had passed around the two right bicuspid teeth, but the movement of the jaw had fortunately released one tooth and the wire hung on the other. Had the wire been fastened around each tooth singly and firmly and then have been twisted so as to hold the two bicuspids close together, one must have

*American Journal of Dental Science, new series, Vol. I, p. 171.

been pulled out, for the jaw was broken between them, and in swallowing even the saliva, the back fragment went up and the front of the jaw went down. Had the wire been passed around the wisdom tooth as well as the second bicuspid and also fastened to three or four teeth in front of the fracture it would have held the bone together on that side for several days, but such control is only temporary. This, however, in a young and vigorous patient, might allow the gum to form adhesions and limit the movement of the parts while more efficient support was prepared.

The wire ligature, however, as used in Mr. Seward's case, was not only useless but dangerous, and probably led to the loss of the second bicuspid tooth. Now, there were a dozen dentists in Washington who could have applied the ligature safely as a temporary support.

These mistakes were made four years after my splint had cured the jaw of the Spanish seaman in the Naval Hospital at New York, and his case was well known for the Spanish Government was very complimentary in regard to it. Further, it was more than two years after, my treatment was brought before the "New York Academy of Medicine," and the "Medical Society of the State of New York," and published in their journals. The splints had also been applied by me in the Civil and the Military Hospitals in many difficult cases. In addition to this, the splint had been used for nearly a year in the Confederate Army Hospitals, with astonishing success.*

It received marked attention from their surgeons, and Surgeon-General Samuel Preston Moore,† with his usual judgment, ordered Dr. Bean to Richmond, in order that the splint might be laid before the Army Medical Board.

It was under these circumstances that Dr. Whelan, Chief of Bureau of Medicine and Surgery for the Navy, after assisting and advising in Secretary Seward's treatment, felt it necessary to insist that I should be called to Washington. The fracture was then into that period when according to Paget and other observers, the reparative process should be at work to unite the fragments of the bone, whereas they were still moving about, even the soft parts having no opportunity to form adhesions.

When I saw Mr. Seward on the twelfth day of the injury, his condition was gravely complicated by the terrible events which had passed since I was sent for. The patient had been free from bandages

*E. N. Covey, Richmond Medical Journal, Vol. I, No. 2, February, 1866.

†American Journal of Dental Science, 3d Series, Vol. I, p. 187.

on his jaw for several days, but he was suffering from the loss of blood through the cuts in his neck and face on the night of the 14th, and also from the pain of these recent wounds. I found upon examination that his chin, the front of the jaw, was moving on the compound fractures on each side between the bicuspid teeth. The right fracture, the one discovered by the patient, being so loose, that it allowed the back fragment to rise high above the front one; thus the end of the back one was much exposed, while the separation between the fragments of the left side was so great that it was hardly possible that the blood vessels in the dental canal were not cut off like those on the right side; and the bone also exposed to the air. This, together with the general condition of the patient, left room for fears of necrosis; but as the patient thought he could not bear his artificial teeth in his mouth, I could not set the bone in place without annoyance to him, so he was left to rest until the next day; as shown in case 8.

Enough has already been shown to prove the need of other assistance, in the light of those views before quoted from Malgaigne and Paget; but to afford every possible help to the student to become an adept in prognosis so far as this is attainable without investigation into the views of these authors in their own works, I present some additional remarks upon the graver complications of Secretary Seward's case.

The report, case 8,* made in 1866, shows, without the amplification now given, that Secretary Seward suffered from two compound fractures of the lower jaw which were uncontrollable by the surgeons in Washington, that his right arm was useless (being under treatment for a fracture near the shoulder) and further that on the tenth day, after I had been sent for, but before I reached him, he was subjected to several cuts on the face, jaw and neck, one of which left the right fragments of the jaw unconnected except by the soft parts under the tongue and that he lost much blood. I found him on the twenty-fourth day also suffering from paralysis in the head, face and lip on the right side, and with the fragments of the jaw still left to move about. The duct of the right parotid was severed and the saliva

*See N. Y. Med. Jour., Vol. iv; Dental Cosmos, Vol. viii, p. 529; American Jour. Dental Science, 3d series, Vol. 2; British Jour. Dental Science, 1866; and Heath's Injuries and Diseases of the Jaws.

flowed through the right fracture· He was sixty-five years old, and was mentally overworked especially during the war. At the time of the accident (April, 1865) the state of the country was critical, and the transfer of the executive power, through the death of President Lincoln, to Andrew Johnson, greatly increased his responsibility as Secretary of State and this while his son, the assistant Secretary lay in imminent danger through the wounds inflicted upon him. Mrs. Seward was seriously ill at the time I removed the Secretary's first splint, and when I took off the second he had buried his wife. In view of what I have already quoted from Malgaigne and Paget, it would be superfluous to explain further, for the mere tyro in surgery may see that going into Mr. Seward's case, as I did, it would not be fair to charge even an unfortunate result to the insufficiency of my treatment, for everything was unfavorable. The fact is, however, that my splints held the fragments of the jaw perfectly. That the jaw united in both fractures although the right did not ossify, but the jaw was used from the time the first splint was removed, and continued to improve so that in a letter to me dated March 29, 1866, Secretary Seward, after speaking of the removal of the loose tooth, said "Since I had the pleasure of seeing you in New York, the soreness of the part has diminished and the whole jaw moves quite well and firmly. Thus at last I begin to regard my cure in that respect complete."

In April, 1867, Baron Gerolt, the minister of Prussia, in writing to me in reference to my pamphlet and the electrotypes explaining my treatment of fractures of the jaw which he had forwarded to his Government; and also to medical authorities in Berlin, closed his letter as follows; " Mr. Seward's lower jaw seems to work admirably well for all purposes."

Mr. Seward's case was never used by me except in connection with others to illustrate my treatment with *Interdental Splints* consequently all these cases may be found with his by any one who wishes to consult them in reference to the splints, of which the illustrations and explanations are now submitted to the readers of the Independent Practitioner.

### INTERDENTAL SPLINTS.

In the year 1840, when treating the first fractured lower jaw placed in my care, I found treatment by bandages unreliable. For, while the muscles tend to displace the bone, bandages frequently increase the difficulty; especially when swelling sets in through their pressure. They also, by interfering with the circulation, tend to prevent union. Teeth, loosened by the injury, are left unsupported, and the motions of the jaw, cheeks and lips painfully restricted.

Of the contrivances invented to supplement bandages, many were even more objectionable, and little improvement has been made in general treatment up to the present time. Having successfully used interdental splints, in many cases which had proved unmanageable under the usual treatment, I am convinced that they are superior to all other appliances.

When a well adapted splint is on the teeth and gum, the other parts around the bone are, to a great extent, a counter support to the splint. Thus the broken jaw, together with any teeth loosened by the injury, is held securely in place, until the fractured bone is re-united and the teeth become firm. Meanwhile the motions of the jaw are in most cases unrestricted and the cheeks and lips always left free.

On February 12th, 1861, I applied a "hard vulcanized rubber splint" to the fractured jaw of a seaman in the United States Naval Hospital, and from the vulcanite splints used by me since that time, I selected three which show all that is essential to hold any fractured lower jaw in place.

The fourth, a metal splint, is sufficient for the treatment of most cases, and can be applied by surgeons and country practitioners, who can also treat most cases of fracture with rubber splints, if assisted by the neighboring dentist.*

The radical and distinctive feature of these splints is, that, when suitable teeth are in the mouth, nothing is required on the outside, and the patient may move about. In the use of these splints fractures of the lower jaw are divided into two distinct classes; first, those in which the teeth and gum of the fractured jaw are alone used to control the fractured bone, and the jaw is allowed to move naturally; second, those in which the splint is fitted to both the upper and lower teeth, the jaw being held still; but no bandage is used around the head.

To apply these splints the fractured jaw should, if possible, be set and held by ligatures around the teeth while an impression of the

*The splints were described in a paper read before the New York Academy of Medicine, June 1st, 1864.

teeth and gum is taken in pure warm wax confined in a cup like No. 4 splint; the plaster cast from the impression will then be precisely what is required to mould the splint. If the bone cannot be held in place an impression may be taken of the teeth in the best attainable position, the plaster cast then separated where necessary and the parts set in place; a cast of the upper teeth will guide in putting these parts of the lower cast in place.

Fig. 1 represents the inner surface of a splint which incloses all the teeth and part of the gum of the lower jaw, and merely rests against the upper teeth when the jaws are closed. This splint is adapted to the treatment of all cases which have teeth in both fragments.

FIG. 1.

The holes marked A go through the top of the splint for the purpose of syringing the parts within with warm water during treatment. The dark round spots in all the cuts represent holes for similar purposes.

*The angles of the jaw tend outward, when the jaw is fractured through the body.* It is therefore necessary that the splint should go down and extend back as far on the outside as the muscles admit, especially on the short fragment, if there is much difference between them. The parts near the external oblique line are so formed that the splint can be fitted to them perfectly. The outer ends of the splint can be fitted to them perfectly. The outer ends of the splint should be quite thick, so that they may be well rounded.

I have generally used this splint without any fastenings, but in children or even adults it is sometimes advisable to secure it by packthread or wire, or by screws passing into or between the teeth, or by the wings and band of Fig. 4.

When screws are used to hold any rubber splint fast on the teeth, metal nuts must be imbedded in the splint, for the screws to work in.

Small openings should be made opposite particular teeth, to observe how the jaw stands in the splint. This is important in all splints.

Fig. 2 Shows a splint for cases in which it is found impracticable to hold the fragments together, except by keeping the fractured bone still; this splint, in addition to fitting the teeth and gum of the lower jaw, must also inclose the upper teeth, as shown in the cut, where screws may be seen opposite both lower and upper teeth.

By this arrangement the fragments of the lower jaw are secured not only relatively to each other, but also to the upper jaw.

This splint is therefore adapted to the treatment of *all fractures back of the teeth*, whether in the body, the rami, or their terminations. In these cases the splint may be cut away in front, and extended across roof of the mouth, when there are upper and lower back teeth to fasten to, and thus give as much room as possible to speak and eat through. Opening the teeth a quarter or three-eighths of an inch would not have any bad effect on the position of the fragments, even if the jaw were broken through the necks of both condyles, as the parts near the fractures would move but little and the back of the jaw could be raised high enough to keep the broken surfaces in contact. Even if the neck of one side only were broken, the lower part could be kept firmly up against the fragment above.

When the jaw is held fast to the upper teeth, especially when wings project through the lips, passages should be cut through the sides of the splint, where the absence of teeth or separation of the jaws gives a chance for the saliva from the parotid glands to enter the mouth, otherwise it may overflow at the lips.

FIG. 3.

F, upper wing. G, lower wing. H, mental band to hold the jaw up in the splint. I, neck strap to keep the band back. K, balance strap to hold the cap in place.

Fig. 3 shows the wings for cases having no teeth in either jaw—the ends of the wings within the mouth being imbedded in a vulcanite splint similar in principle to that of Fig. 2.

Wings made of steel or of iron may be quite light. They should have small holes every half inch to hold the strings, lacing, etc. The arch of the wings should be high enough to give the lower lip room to go well up. The wings for each side of the jaw are in one piece, and the parts within the mouth pass back in the line of the upper gum. They are thinned down and pierced with holes, that the rubber in which they are imbedded may hold them firmly.

The tape strings pass from the cap inside and under the upper wings, then up between them and the tape lacings which keep the strings from slipping to the cap whence they started. The mental band (which is only one thickness of linen,) passes up between the sides of the lower jaw and the wings where it is tied by the strings, which pass through the holes. The band is cut off to show this; but when worn it should be turned down on the outside and pinned just below the wings. The neck strap should be sewed to the mental band on one side and pinned on the other, and worn tight enough to keep the band from slipping forward over the chin.

The jaw and splint are supported by the cap forward of its centre. This is counterbalanced by the elastic strap which passes from the back of the cap down around an unelastic and much heavier strap, extending across and fastened to the shoulders by elastic ends. The balance strap returns to the cap and is buckled tight enough to hold the jaw up. At night it may be slackened to do this, with the neck flexed. It slides on the shoulder strap as the head incline to either side.

By this arrangement the splint is a resting place for the broken, jaw, while the wings give firm attachment to appliances which hold the jaw up with the least possible pressure upon the external parts, as the wings need not press either against the jaw or the zygomas.

Fig. 4 represents a splint devised in 1863, for the use of practitioners out of the reach of a dentist, and for hospital use. This splint is made of cast tin, and is applied with a lining of gutta-percha. It is in the shape of an impression cup, and seven sizes are kept ready for use from which one can be selected for the broken jaw. The wings are of malleable iron, tinned to prevent rusting and for more readily soldering. Three sizes are sufficient to select from.

FIG. 4.

G, wing of malleable iron, projecting, with its fellow, from the splint to which they are soldered. H, mental or splint band, with the end left up to show the manner of tying it. I, neck strap. The mental band is made of linen or any thin material.

The splint has a handle in front, that it may be used as a cup to take the impression of the jaw—the holes being useful to allow a small probe to be pressed through the wax down to the teeth, thus allowing air to enter to facilitate the removal of the impression, and when in use as a splint giving entrance to warm water, thrown from a syringe, to keep the parts clean.

The splint should be made to fit well by bending, cutting off the edges and rounding them up smooth. When a tooth projects so as to keep the splint from fitting, a hole may be cut to let the tooth through, if the metal cannot be hammered out. This should all be done before taking the impression, as a well fitted cup assists greatly in this important matter.

After the *cast* is obtained, the handle in front should be cut off, and the wings, *if needed*, soldered on, care being taken that their edges are clear of the corners of the mouth, when *open*. Warm gutta-percha should then be placed in the splint, pressed down on the cast, and, after cooling in water, dig out the softened plaster.

This splint has the advantage of being easier of application, and can be applied in much shorter time than a rubber splint, especially if the fractured bone can be set and held by ligatures firmly enough to bear the pressure of the warm gutta-percha, for the splint can then be at once applied to the teeth and the gutta-percha closing around them, the bone will keep in place without other fastenings.

When the fragments of the jaw cannot be held firmly enough to bear the pressure of warm gutta-percha without displacement, plaster of Paris would hold the jaw securely in the splint for a long time. In these methods the ligatures are left on.

*THE END.*

## Figures and Tables

**FIG. 1. f1:**
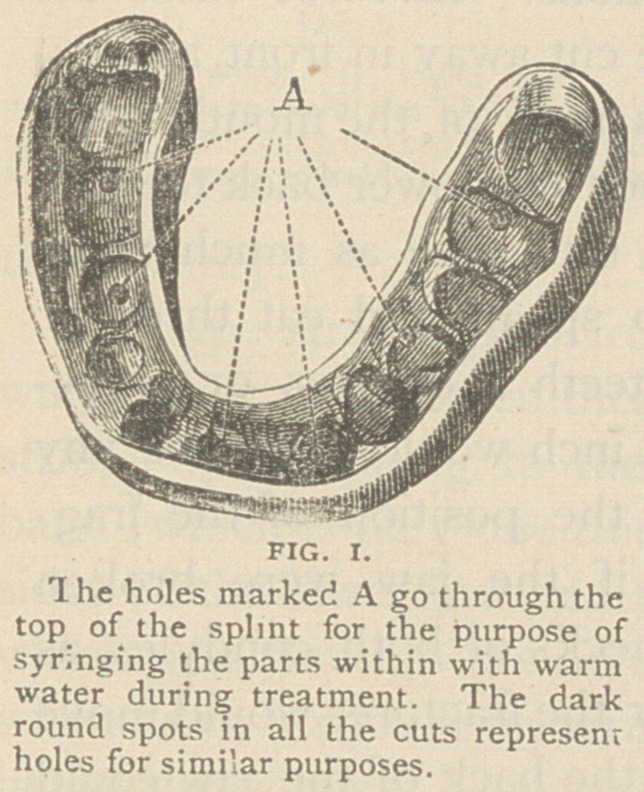


**FIG. 2. f2:**
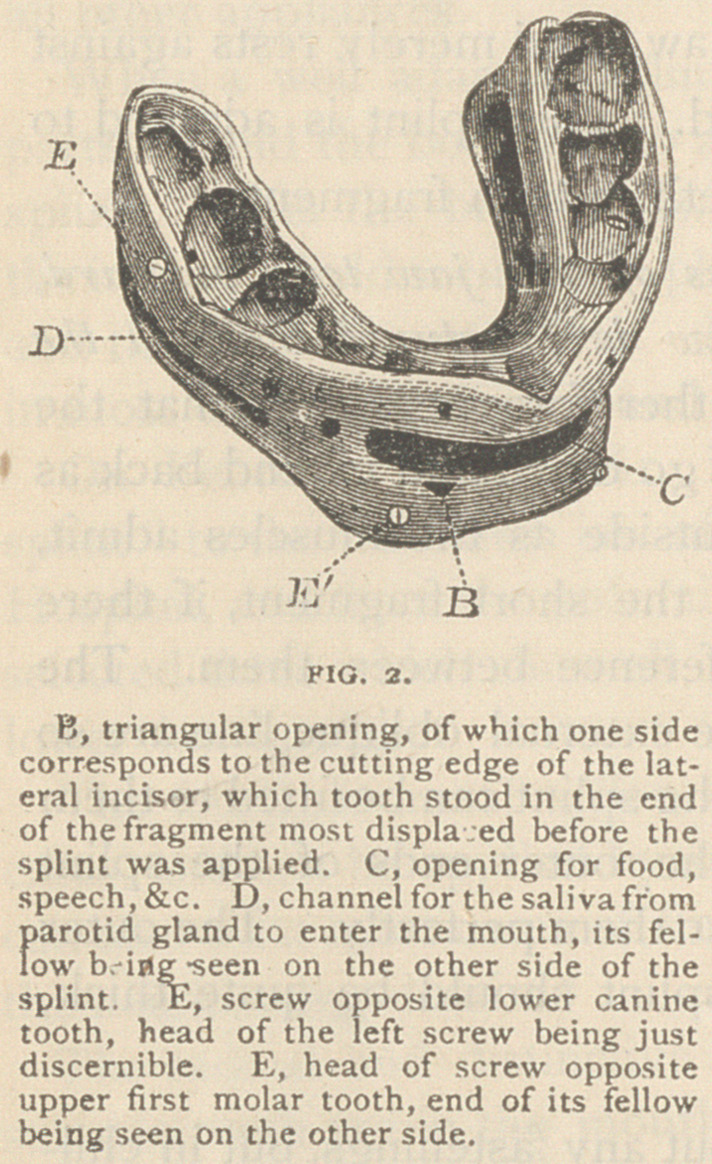


**FIG. 3. f3:**
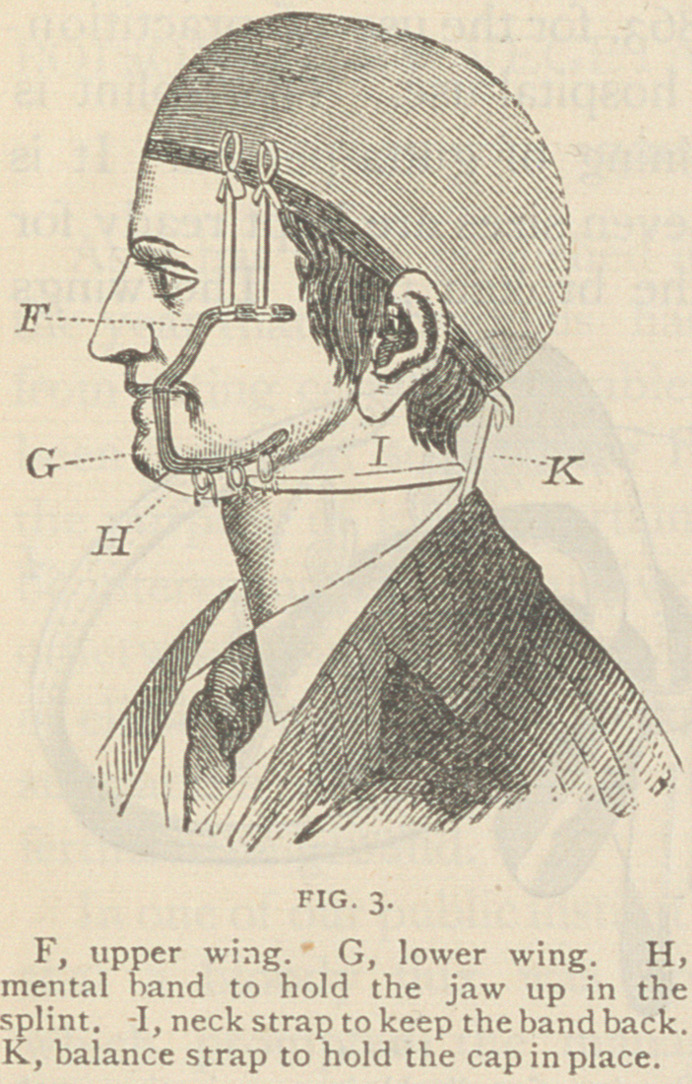


**FIG. 4. f4:**